# Partial substitution of red or processed meat with plant-based foods and the risk of cardiovascular disease

**DOI:** 10.1007/s10654-025-01232-x

**Published:** 2025-05-12

**Authors:** Meri Simojoki, Niina E. Kaartinen, Mirkka Maukonen, Kennet Harald, Heli Tapanainen, Demetrius Albanes, Johan G. Eriksson, Pekka Jousilahti, Seppo Koskinen, Anne-Maria Pajari, Satu Männistö

**Affiliations:** 1 Department of Public Health, Finnish Institute for Health and Welfare (THL), P.O. Box 30, FI-00271 Helsinki, Finland; 2 Division of Cancer Epidemiology and Genetics, National Cancer Institute, National Institutes of Health (NIH), Bethesda, MD USA; 3 Department of General Practice and Primary Health Care, University of Helsinki and Helsinki University Hospital, Helsinki, Finland; 4 Folkhälsan Research Center, University of Helsinki, Helsinki, Finland; 5 Department of Obstetrics and Gynaecology and Human Potential Translational Research Programme, Yong Loo Lin School of Medicine, National University of Singapore, Singapore, Singapore; 6 Agency for Science, Technology and Research, Singapore Institute for Clinical Sciences (SICS), A*STAR, Singapore, Singapore; 7 Department of Food and Nutrition, University of Helsinki, Helsinki, Finland

**Keywords:** Cardiovascular disease, Fruits, Legumes, Vegetables, Whole grain cereals

## Abstract

A shift towards more plant-based diets may promote human and planetary health. This modelling study aimed to assess the impact of moderate partial substitution of red or processed meat with plant-based foods on cardiovascular disease (CVD) risk. We used pooled data from five Finnish cohorts (42,868 participants aged ≥ 25 years, 78% men). Median follow-up time was 12.7 years, with 11,031 incident CVD cases. Diet was assessed by a validated food frequency questionnaire. We modelled substitutions of red meat (100 g/week) or processed meat (50 g/week) with corresponding amounts of plant-based foods. Cohort-specific hazard ratios (HR) and 95% confidence intervals (CI) were calculated using Cox proportional hazards multivariate models adjusted for relevant confounding factors and pooled together using a random effects model. There was a suggestive reduction in CVD risk in men when processed meat was substituted with vegetables or the combination of plant-based foods (HR 0.99, 95% CI 0.99–1.00, P < 0.05). In women, there was an increase in CVD risk when red meat was substituted with legumes (HR 1.10, 95% CI 1.01–1.20, P < 0.05). However, when all cohorts were followed for the same length of time (7.9 years), several plant-based foods reduced CVD risk and none of them increased the risk when partially replacing red or processed meat. Even a small, easily implemented change towards a more plant-based diet may contribute to cardiovascular health at the population level. These findings support global strategies towards healthy and environmentally sustainable diets.

**Supplementary Information:**

The online version contains supplementary material available at 10.1007/s10654-025-01232-x.

## Introduction

Cardiovascular diseases (CVD) are the leading cause of death globally [1]. High consumption of red or processed meat has been associated with an increased CVD risk, whereas plant-based foods may lower the risk [2]. A shift from animal-based diets to more plant-based diets is likely to reduce the risk of CVD and, moreover, promote environmental sustainability [3].

The diet of the Finnish adult population improved between 1997 and 2017 [4]. In men and women, the consumption of vegetables, fruits, berries, legumes, nuts, and seeds increased between 1997 and 2017, while men’s consumption of red and processed meat decreased between 2012 and 2017. However, 93% of men and 60% of women exceed the current Finnish and Nordic nutrition recommendation of 350 g/week for red and processed meat [5–7]. In addition, only 14% of men and 22% of women consume vegetables, fruits, and berries more than the recommended 500 g/day. Therefore, a shift to a more plant-based diet is necessary, and partial substitution of animal-based foods with plant-based alternatives can contribute to this shift.

Thus far, a few modelling studies have assessed the impact of partial substitution of red or processed meat with plant-based foods on the risk of CVD in large population-based samples. These studies included Danish adults aged 50–64 years [8], US adults aged 30–75 years [9–12], and Singapore residents aged 21–75 years [13]. Previous studies suggest that partial substitution of red or processed meat with vegetables, potatoes, nuts, legumes, soya, whole grains, or combined plant protein sources may reduce the risk of CVD. However, only one study included vegetables as substitutes [8], and none included fruits. Most studies replaced one serving of meat per day [9–12], and only two studies replaced one serving per week or 3–4 servings per week [8, 11]. In addition, one study used isocaloric substitution [13].

A small change towards a more plant-based diet may be easier to implement in real life and can also lead to more permanent dietary changes. Substituting 100 g of red meat and 50 g of processed meat per week with plant-based foods would reflect such a small change. These amounts correspond to the daily consumption or serving size of red and processed meat in Finland [5, 6]. These amounts also reflect the recommended serving sizes for plant-based foods [6].

Using pooled data from five large Finnish cohorts, we aimed to assess the impact of moderate partial substitution of red meat (100 g/week) or processed meat (50 g/week) with plant-based foods (legumes, vegetables, fruits, whole grain cereals, or a combination of these) on CVD risk in Finnish adults.

## Methods

### Participants

We used pooled data from five Finnish cohorts conducted at the Finnish Institute for Health and Welfare (THL): the Alpha-Tocopherol, Beta-Carotene Cancer Prevention Study (ATBC) [14], the Health 2000 Survey (Health 2000) [15], the Helsinki Birth Cohort Study (HBCS) [16], the Dietary, Lifestyle, and Genetic Determinants of Obesity and Metabolic Syndrome 2007 Study (DILGOM 2007) [17], and the National FINRISK 2012 Study (FINRISK 2012) [18].

Each cohort included a health examination and self-administered questionnaires. Diet was assessed at baseline using a validated food frequency questionnaire (FFQ), and CVD cases (coronary heart disease (CHD) or stroke) were confirmed by national health registers. We included a total of 42,868 participants with an approved FFQ and without prevalent CVD at baseline in this study.

This study was conducted in accordance with the Declaration of Helsinki. Each cohort study followed the ethical standards of its time and was approved by the relevant ethics committee [14–18]. For example, the National FINRISK 2012 Study was approved by the Ethics Committee of the Hospital District of Helsinki and Uusimaa (162/13/03/00/2011, 20 September 2011). All participants provided written informed consent.

### Dietary assessment

In each cohort, diet was assessed using a validated food frequency questionnaire [19–22]. The FFQs covered the habitual diet over the past 12 months. The foods inquired included the most used foods in Finland based on the National FinDiet Surveys conducted since 1982 [5]. In the ATBC study, the FFQ included 276 food items and mixed dishes. The usual frequency of consumption was recorded according to daily, weekly, or monthly consumption, based on 3–5 portion sizes depicted in a portion size picture booklet. In the other cohorts, the FFQ included approximately 130 food items and mixed dishes whose consumption was recorded in nine frequency categories (from ‘never or seldom’ to ‘at least six times a day’) using fixed portion sizes.

Participants filled FFQs at home (ATBC, Health 2000, FINRISK 2012) or at the study site (HBCS, DILGOM 2007). Incomplete FFQs (several blank food item lines) were excluded. Implausible energy intake at both ends of the energy intake distribution also led to the exclusion of FFQs (ATBC: daily energy intake < 1000 kcal or > 5000 kcal; Health 2000: daily energy intake < 600 kcal or > 7000 kcal; HBCS, DILGOM 2007 and FINRISK 2012: 0.5% extremes in energy intake distribution by sex [23]). Of the FFQs given to participants, 92% were approved.

Food consumption and energy intake were calculated using the Finnish National Food Composition Database Fineli® and an in-house software [24]. Red meat included beef, pork, lamb, and game (Online Resource 1). Processed meat included sausages and cold cuts. Legumes included all types commonly used in Finland, such as green peas, beans, and soya. Vegetables included all vegetables (except legumes and potatoes), as well as nuts and seeds, whose consumption is low in Finland [5]. Fruits included all fruits and berries. Whole grain cereals included rye, oat, and barley, the combination of which has been shown to correspond well (r = 0.99) to total whole grain intake in Finnish adults [25]. Wheat was not included in whole grain cereals because we were unable to separate whole wheat from refined wheat. Standard recipes in the database were used in the calculations to decompose mixed dishes into their ingredients (e.g. uncooked red meat, rye flour).

### Cardiovascular disease ascertainment

Cases of CVD (CHD or stroke) were confirmed by national administrative registers on hospitalizations or causes of death. The unique personal identity code of Finnish citizens was used to combine the participants and the data in the registers.

For non-fatal CHD events, the International Classification of Diseases (ICD) codes were ICD-10: I200, I21, I22; ICD-8 or ICD-9: 410, 4110. For fatal CHD events, ICD-codes were ICD-10: I20-I25, I46, R96, R98; ICD-8 or ICD-9: 410–414, 798 (except 7980A). For non-fatal and fatal stroke events, ICD-codes were ICD-10: I61, I63 (except I636), I64; ICD-9: 431, 4330A, 4331A, 4339A, 4340A, 4341A, 4349A, 436; ICD-8: 431 (except 43101, 43191), 433, 434, 436.

Participants with prevalent CVD at baseline (n = 1690) were excluded. The cohorts were followed until the end of 2013 (HBCS), 2015 (Health 2000), 2016 (ATBC), or 2019 (DILGOM 2007, FINRISK 2012). The median follow-up time was 12.7 years, with 11,031 incident CVD cases.

### Potential confounding variables

Self-administered questionnaires collected information on socioeconomic status, health, and lifestyle. These variables were harmonized across the cohorts. The total years of education were divided into birth cohort-specific tertiles, except for ATBC study, where ‘highest education’ included those with more than upper secondary education. Smoking habits were divided into three categories (never, former, current) as well as leisure-time physical activity (passive, somewhat active, active). The use of alcohol was expressed as ethanol (g/day). Hormone replacement therapy was classified into two categories for women (ever, never).

In the health examination, trained study nurses measured weight, height, and blood pressure (systolic and diastolic, mmHg), and took blood samples according to standard protocols [26]. Body mass index (BMI) was calculated as weight in kilograms divided by the square of height in meters (kg/m^2^). Total serum cholesterol (mmol/l) was analysed from the blood samples.

### Statistical analysis

We modelled the substitution of 100 g/week of red meat or 50 g/week of processed meat with similar amounts of legumes, vegetables, fruits, whole grain cereals, or a combination of these. Red meat and processed meat were studied separately as they may have a different impact on the risk of CVD [2]. We conducted substitution analyses by sex, as the interaction analysis suggested that the results differ for men and women. Baseline characteristics of the participants were calculated in different cohorts as medians and interquartile ranges (IQR) for continuous variables and as proportions for categorical variables.

Initially, we calculated the associations between CVD risk and each food group used in the substitution analysis according to their consumption quintiles (Online Resource 2). Cohort-specific hazard ratios (HR) and 95% confidence intervals (CI) were calculated using Cox proportional hazards multivariate models adjusted for relevant confounding factors. The pooled HRs were estimated from the cohort-specific HRs weighted by the inverse of their variances using a random effects model [27]. The heterogeneity between the pooled cohorts was tested by Q-statistics.

Substitution analyses were based on a leave-one-out model [28]. The substitution models included a substitute (legumes, vegetables, fruits, whole grain cereals, or a combination of these) as a separate variable, as well as a sum variable consisting of the substitute and the substituted food group (red or processed meat) (Online Resource 3). Derived hazard ratios and 95% confidence intervals showed an estimated CVD risk associated with an increase in consumption of plant-based substitutes and a concomitant decrease in consumption of red or processed meat.

We selected potential confounding factors for the analyses based on the literature. The first model was adjusted for age and energy intake. The second model was further adjusted for education, smoking, leisure-time physical activity, body mass index, systolic blood pressure, diastolic blood pressure, total serum cholesterol, hormone replacement therapy (for women), and alcohol consumption. We used alcohol as a continuous variable instead of a categorical variable, because the results remained virtually unchanged when alcohol was used as a categorical variable.

We also conducted additional analyses in which other food variables were considered as confounding factors. We created a meat variable that included fish and poultry as well as red or processed meat depending on which food (red or processed meat) was substituted. We then added this variable to the adjustments when modelling the substitution of 100 g/week of red meat and 50 g/week of processed meat.

For health reasons, it is recommended that the consumption of red meat (including red meat in products and processed foods) should be low and not exceed 350 g/week ready-to-eat (cooked) weight [6, 7]. Processed red meat should be as low as possible. For environmental reasons, the consumption of red meat should be considerably lower than 350 g/week. Therefore, we conducted additional analyses to assess the potential change in CVD risk with larger substituted amounts. We increased the substituted amounts to 100 g per day for red meat and 50 g per day for processed meat.

In sensitivity analyses, we further excluded participants who consumed red meat less than 100 g/week and processed meat less than 50 g/week from the analyses. In another sensitivity analysis, participants diagnosed with CVD in the first two years of follow-up were excluded due to concerns about reverse causation.

Since the diet may change over time, we conducted additional analyses in which all cohorts were followed for the same length of time. The length of follow-up corresponded to the follow-up time of the National FINRISK 2012 Study, which was the most recent cohort and had the shortest follow-up time. In these analyses, the follow-up time was 7.9 years with 4421 incident CVD cases.

Statistical analyses were conducted using R statistical software version 3.6 [29]. R package meta was used for pooling of hazard ratios [30].

## Results

The pooled data included 42,868 participants who were at least 25 years of age at baseline, and 78% were men (Table 1). The median baseline age of participants ranged from 50 years (Health 2000) to 60 years (HBCS) (Table 2). The ATBC study included only male smokers, who were also less likely to have high education (6% vs. 34–36%) and less likely to be physically active in their leisure time (6% vs. 17–31%) compared to participants in other cohorts. Participants in the ATBC study also tended to consume more alcohol, processed meat, and whole grain cereals, but fewer legumes, vegetables, and fruits compared to participants in other cohorts. The median consumption of legumes was low in all cohorts (4–10 g/day).

**Table 1:** Cohorts included in the pooled analyses of partial substitutions of red meat or processed meat with plant-based foods and the risk of cardiovascular diseases (CVD)

Cohort	Baseline years	Baseline age, years	Final number of participants^a^	Number of men (%)	Median follow-up time, years	Number of incident CVD cases
ATBC^b^	1984–1988	50–69	25,850	25,850 (100%)	15.8	9823
Health 2000^c^	2000–2001	30–99	5667	2473 (44%)	15.0	775
HBCS^d^	2001–2004	56–69	1917	870 (45%)	11.1	96
DILGOM 2007^e^	2007	25–74	4755	2162 (45%)	12.6	228
FINRISK 2012^f^	2012	25–74	4679	2096 (45%)	7.8	109
Total	–	–	42,868	33,451 (78%)	12.7	11,031

^a^Including participants with an approved food frequency questionnaire and without prevalent CVD at baseline

^b^The Alpha-Tocopherol, Beta-Carotene Cancer Prevention Study [14]

^c^The Health 2000 Survey [15]

^d^The Helsinki Birth Cohort Study [16]

^e^The Dietary, Lifestyle and Genetic Determinants of Obesity and Metabolic Syndrome 2007 Study [17]

^f^The National FINRISK 2012 Study [18]

**Table 2:** Baseline characteristics of the participants included in the pooled analyses by cohorts (medians and interquartile ranges or percentages)

	ATBC^a^	Health 2000	HBCS	DILGOM 2007	FINRISK 2012
*Confounding factors*					
Age, years	57.0 (8.0)	50.0 (21.0)	60.0 (4.0)	52.9 (22.1)	53.0 (24.0)
Energy, MJ/day	10.9 (4.0)	9.1 (3.9)	8.7 (4.1)	9.9 (4.6)	8.9 (4.1)
Alcohol, g/day	11 (23)	2 (7)	5 (11)	4 (9)	4 (9)
High education^b^, %	6	34	34	36	34
Current smoker, %	100	26	23	17	17
Active in leisure time^c^, %	6	17	26	28	31
Body mass index, kg/m^2^	26.0 (4.8)	26.2 (5.9)	26.9 (5.4)	26.1 (5.6)	26.2 (6.0)
Systolic blood pressure, mmHg	140 (26)	131 (28)	144 (27)	131 (24)	130 (23)
Diastolic blood pressure, mmHg	88 (14)	81 (15)	88 (14)	79 (15)	80 (15)
Total serum cholesterol, mmol/l	6.2 (1.5)	5.9 (1.4)	5.9 (1.3)	5.2 (1.3)	5.3 (1.4)
Hormone replacement therapy (women), ever %	-	32	68	16	15
*Substitution variables*					
Red meat, g/day	65 (39)	73 (50)	60 (49)	73 (58)	65 (51)
Processed meat, g/day	60 (58)	36 (48)	30 (39)	40 (49)	37 (48)
Legumes, g/day	4 (5)	9 (9)	8 (9)	10 (10)	10 (10)
Vegetables^d^, g/day	95 (84)	219 (187)	247 (203)	265 (219)	230 (190)
Fruits, g/day	108 (117)	159 (207)	220 (273)	216 (243)	156 (184)
Whole grain cereals^e^, g/day	100 (85)	63 (57)	58 (49)	83 (62)	74 (64)

^a^Men 100%

^b^The total number of school years was divided into birth cohort specific tertiles, except for the ATBC study, where ‘highest education’ included those with more than upper secondary education

^c^Exercise at least three hours a week or competition or other heavy sports several times a week

^d^Nuts and seeds included, legumes and potatoes excluded

^e^Rye, oat, and barley, the combination of which has been shown to correspond well (r = 0.99) to total whole grain intake in Finnish adults[25]

Red meat and processed meat consumption were not associated with CVD risk in the pooled data (Online Resource 2, model 2). Vegetable consumption was associated with a 15% lower CVD risk in the participants of the highest consumption quintile compared to the lowest quintile (HR 0.85, 95% CI 0.76–0.94, P_trend_ < 0.001, model 2), while legumes, fruits, and whole grain cereals did not contribute to the risk (P_trend_ > 0.05, model 2).

In men, substituting 50 g/week of processed meat with vegetables (HR 0.99, 95% CI 0.99–1.00, P = 0.040) or the combination of plant-based foods (HR 0.99, 95% CI 0.99–1.00, P = 0.031) reduced CVD risk (Table 3, model 2). In women, substituting 100 g/week of red meat with legumes (HR 1.10, 95% CI 1.01–1.20, P = 0.027) increased CVD risk. We observed no notable heterogeneity between the cohorts.

**Table 3:** Pooled associations between partial substitutions of red meat (100 g/week) or processed meat (50 g/week) with legumes, vegetables, fruits, whole grain cereals, or a combination of these, and cardiovascular disease risk in men and women with a median follow-up of 12.7 years

	Men			Women		
	Model 1^a^	Model 2^b^		Model 1^a^	Model 2^b^	
	HR (95% CI)^c^	HR (95% CI)^c^	P_het_^d^	HR (95% CI)^c^	HR (95% CI)^c^	P_het_^d^
*Substitution of red meat (100 g/week) with*						
Legumes^e^, 100 g/week	0.97 (0.90, 1.05)	0.98 (0.92, 1.04)	0.39	1.09 (1.00, 1.19)	1.10 (1.01, 1.20)*	0.50
Vegetables^f^, 100 g/week	0.98 (0.97, 0.99)**	0.99 (0.98, 1.00)	0.63	0.98 (0.96, 1.01)	0.99 (0.96, 1.01)	0.66
Fruits, 100 g/week	1.00 (0.99, 1.00)	1.00 (0.99, 1.01)	0.81	0.98 (0.95, 1.00)	0.98 (0.95, 1.01)	0.79
Whole grain cereals^g^, 100 g/week	1.00 (0.99, 1.01)	1.00 (0.99, 1.01)	0.92	0.97 (0.93, 1.01)	0.98 (0.94, 1.02)	0.95
Legumes, vegetables, fruits, and whole grain cereals, 100 g/week	0.99 (0.98, 1.00)*	1.00 (0.99, 1.00)	0.77	0.98 (0.95, 1.00)	0.98 (0.96, 1.01)	0.75
*Substitution of processed meat (50 g/week) with*						
Legumes^e^, 50 g/week	0.98 (0.95, 1.01)	0.98 (0.96, 1.01)	0.62	1.07 (0.99, 1.17)	1.08 (0.99, 1.17)	0.048
Vegetables^f^, 50 g/week	0.99 (0.98, 1.00)**	0.99 (0.99, 1.00)*	0.54	0.98 (0.97, 1.00)*	0.99 (0.97, 1.00)	0.062
Fruits, 50 g/week	0.99 (0.98, 1.00)*	0.99 (0.99, 1.00)	0.20	0.98 (0.97, 1.00)*	0.99 (0.97, 1.00)	0.053
Whole grain cereals^g^, 50 g/week	0.99 (0.98, 1.00)	1.00 (0.99, 1.00)	0.89	0.98 (0.96, 1.01)	0.99 (0.97, 1.01)	0.18
Legumes, vegetables, fruits, and whole grain cereals, 50 g/week	0.99 (0.98, 1.00)**	0.99 (0.99, 1.00)*	0.50	0.98 (0.97, 1.00)*	0.99 (0.97, 1.00)	0.064

^a^Model 1: adjusted for age (years, continuous) and energy intake (kJ/day, continuous)

^b^Model 2: adjusted for Model 1 + education (tertiles by birth year), smoking (never, former, current), leisure-time physical activity (passive, somewhat active, active), body mass index (kg/m^2^, continuous), systolic blood pressure (mmHg, continuous), diastolic blood pressure (mmHg, continuous), total serum cholesterol (mmol/l, continuous), hormone replacement therapy (only in women) (ever, never), alcohol consumption (as ethanol, g/day, continuous)

^c^Hazard ratio and 95% confidence interval

^d^P for heterogeneity from Q-statistics between pooled cohorts. Adjusted as Model 2

^e^Daily consumption of red and processed meat is much higher than that of legumes in Finnish adults, and therefore these results should be interpreted with caution [5, 28]

^f^Nuts and seeds included, legumes and potatoes excluded

^g^Rye, oat, and barley, the combination of which has been shown to correspond well (r = 0.99) to total whole grain intake in Finnish adults [25]

^*^P < 0.05, **P < 0.01

When other types of meat were considered as confounding factors, the results remained mainly unchanged. In men, substituting 50 g/week of processed meat with the combination of plant-based foods (HR 0.99, 95% CI 0.99–1.00, P = 0.15) no longer reduced CVD risk (model 2, data not shown). In women, substituting 50 g/week of processed meat with legumes (HR 1.06, 95% CI 1.02–1.10, P = 0.002) increased CVD risk.

Increasing the substitutions to 100 g/day for red meat and 50 g/day for processed meat, associations with CVD risk strengthened (Online Resource 4). In men, substituting 50 g/day of processed meat with vegetables (HR 0.93, 95% CI 0.91–0.96, P < 0.001), fruits (HR 0.95, 95% CI 0.92–1.00, P = 0.027) or the combination of plant-based foods (HR 0.96, 95% CI 0.92–1.00, P = 0.035) reduced CVD risk (model 2). In women, substituting 100 g/day of red meat with legumes (HR 1.97, 95% CI 1.07–3.61, P = 0.029) increased CVD risk (model 2).

Excluding participants consuming less than 100 g/week of red meat and 50 g/week of processed meat did not change the results for men (Online Resource 5, sensitivity analysis 1, model 2). In women, however, substituting processed meat partially with legumes increased CVD risk. Excluding those diagnosed with CVD in the first two years of follow-up did not alter the findings (Online Resource 5, sensitivity analysis 2, model 2).

With a shortened follow-up time of 7.9 years, several plant-based foods reduced CVD risk when partially replacing red or processed meat. In men, substituting 50 g/week of processed meat with legumes (HR 0.96, 95% CI 0.93–1.00, P = 0.033) or vegetables (HR 0.99, 95% CI 0.99–1.00, P < 0.001) reduced CVD risk (Online Resource 6, model 2). In women, substituting 100 g/week of red meat with fruits (HR 0.96, 95% CI 0.93–1.00, P = 0.024) or the combination of plant-based foods (HR 0.97, 95% CI 0.94–1.00, P = 0.038) also reduced CVD risk. Further, in women, substituting 50 g/week of processed meat with vegetables (HR 0.98, 95% CI 0.96–0.99, P < 0.01), fruits (HR 0.98, 95% CI 0.96–0.99, P < 0.01), or the combination of plant-based foods (HR 0.98, 95% CI 0.96–0.99, P < 0.01) reduced CVD risk. The results were consistent with substitutions of 100 g/day for red meat and 50 g/day for processed meat, leading to a reduction in CVD risk from 7% to 23% in men and 15% to 23% in women (data not shown).

## Discussion

In pooled data from five large Finnish cohorts, we assessed the impact of moderate partial substitution of red or processed meat with legumes, vegetables, fruits, whole grain cereals, or a combination of these on the risk of CVD. With a maximum follow-up time (median follow-up of 12.7 years), we observed a suggestive reduction in CVD risk in men when substituting 50 g/week of processed meat with vegetables or the combination of plant-based foods, and an increase in CVD risk in women when substituting 100 g/week of red meat with legumes. With a shortened follow-up time of 7.9 years, which was equal to the follow-up time in the most recent cohort, several plant-based foods reduced CVD risk and none of them increased the risk when partially replacing red or processed meat in men and women.

Our results are largely in line with previous modelling studies, where red or processed meat was substituted with various plant-based foods [8–13]. The discrepancies in results between previous studies and our study are probably explained by differences in study design and modelling strategies, as well as the characteristics and food consumption of the study population.

In two previous studies, like in our study, the substituted amounts were moderate (one serving per week), leading to a slight reduction in CVD risk. In a Danish study based on the Danish Diet, Cancer, and Health study, substituting 150 g/week of total red meat (unprocessed and processed red meat) with vegetables reduced the risk of myocardial infarction (MI) in women by 6% [8]. However, when unprocessed red meat and processed red meat were studied separately, only substituting unprocessed red meat with vegetables was associated with MI. In a US study based on six prospective cohorts, substituting one serving per week of unprocessed red meat or processed meat with legumes, whole grains, or nuts decreased the risk of CVD by 1–5% [11].

In most previous studies, like in our additional analyses, the substituted amounts were one serving per day, leading to greater reductions in CVD risk compared to moderate substitutions. In a US study based on the Health Professionals Follow-Up Study, decreases in CHD risk in men were observed when substituting one daily serving of total red meat, unprocessed red meat, or processed red meat with one daily serving of legumes (17–20%), whole grains (38–41%), nuts (11–15%), or combined plant protein sources (nuts, legumes, and soya) (13–17%), and when substituting ≥ 2 servings/week of red meat with ≥ 2 servings/week of soya (33–34%) [9]. Also, in a US study based on the Nurses’ Health Study, substituting one daily serving of red meat with one daily serving of nuts was associated with a 30% lower risk of CHD in women whereas substituting one daily serving of red meat with one daily serving of beans was not associated with the risk [10]. Another US study based on six prospective cohorts showed that substituting one daily serving of unprocessed red meat or processed meat with legumes, whole grains, or nuts decreased the risk of CVD by 9–34% [11]. However, in a US study based on the Atherosclerosis Risk in Communities (ARIC) Study, increasing one daily serving of legumes or nuts at the expense of decreasing one daily serving of red meat or processed meat was not associated with the risk of CHD [12].

In earlier studies, substituting red or processed meat with legumes either decreased CVD risk [9, 11], or was not associated with the risk [10, 12]. In our study, with a maximum follow-up time, legumes increased CVD risk in women when replacing red meat. With a shortened follow-up time, this association attenuated to non-significant. In general, foods included in the substitution analysis should be commonly consumed in the study population [28]. Therefore, this result is likely related to the low consumption of legumes in our cohorts, with a median consumption of 4–10 g per day. Furthermore, the confidence intervals for the results related to the substitutions with legumes were very wide, which also indicates the uncertainty associated with this result. Thus, this finding should be interpreted with caution.

In former studies, fruits were not included in substitution analyses separately. In our study, with a maximum follow-up time, we found no association when partially substituting red or processed meat with fruits. However, with a shortened follow-up time, fruits decreased CVD risk in women when substituting 100 g/week of red meat and 50 g/week of processed meat. Therefore, as a novel finding, also fruits may promote cardiovascular health when substituting red or processed meat.

Previous studies investigated nuts and soya as substitutes for red or processed meat [9–12]. Due to the low consumption of nuts and soya in Finland, they were not studied separately in our study. Instead, nuts were included in vegetables, and soya in legumes.

In earlier studies, whole grains decreased CVD risk when substituting red or processed meat [9, 11]. In our study, there were no associations between moderate substitutions of red or processed meat with whole grain cereals. Differences in the consumption of whole grain cereals in study populations likely explain the differences between studies. In Finland, the consumption of cereals is generally high, and a large part of it is whole grain [5, 31]. Partial substitution of red or processed meat with whole grain cereals did not provide additional benefits in relation to the CVD risk probably because the consumption of whole grain cereals was already high in the pooled cohorts.

We found that with a shortened follow-up time of 7.9 years, several plant-based foods reduced CVD risk and none of them increased the risk when partially replacing red or processed meat in men and women. The differences in results between different follow-up times may be explained by changes in diet over time in Finland [4]. With a shortened follow-up time, the diet probably included more red and processed meat and fewer plant-based foods compared to the maximum follow-up time, leading to stronger associations in modelled substitutions.

We studied moderate dietary changes using 100 g/week for red meat and 50 g/week for processed meat in substitution analyses. We found that these small, easily implemented substitutions can improve cardiovascular health at the population level. Regarding substituted amounts, 100 g of red meat is about the size of a small beef steak (cooked) and 50 g of processed meat is roughly a small sausage or 3–4 slices of cold cuts. For plant-based substitutes, 50–100 g corresponds to 0.5–1 portions of vegetables or 0.5–1 small apples. Such minor weekly dietary changes are probably easy to adopt and can lead to a more plant-based diet.

Although the reduction in CVD risk in this study was suggestive when substituting 100 g/week for red meat and 50 g/week for processed meat with plant-based foods, the public health message is encouraging. Even a slight change towards more plant-based diets may reduce the risk of CVD. A small reduction in the risk of CVD at the population level is important too, as CVD prevalence is high worldwide. In addition, both previous studies and our study suggest that CVD risk is reduced more when substituting larger amounts of red or processed meat with plant-based foods.

Strengths of this study include the use of a large, pooled dataset of five Finnish cohorts, with detailed information on the socioeconomic status, health, and lifestyle of the participants, as well as a long follow-up time. We observed no notable heterogeneity across the pooled cohorts. Another strength is that CVD cases (coronary heart disease or stroke) were confirmed by comprehensive national health registers on hospitalizations or causes of death of the Finnish population. In addition, the diet was assessed with a validated food frequency questionnaire which was developed and updated regularly at the Finnish Institute for Health and Welfare.

This study also has some limitations. Potential misreporting (underreporting or overreporting) and misclassification may be sources of error in self-reported dietary assessment methods. However, energy adjustment in our analyses likely reduced these impacts. Also, as dietary data were only collected at baseline, potential changes in diet over time were not considered. Furthermore, we used a gram-for-gram substitution approach, which, despite energy adjustment, may lead to differences in energy intake. Not using isocaloric substitution, however, likely has a minor impact on the results, as the amounts substituted were very moderate. Additionally, we were not able to consider sleep, ethnicity, and smoking-related details, such as the number of cigarettes per day, as confounding factors. Moreover, our statistical modelling of substitutions may not fully represent real-life dietary changes.

In conclusion, this study highlights the impacts of moderate partial substitution of red or processed meat with plant-based foods on CVD risk in Finnish adults. Substituting 100 g/week of red meat or 50 g/week of processed meat with plant-based foods may reduce CVD risk. Thus, even a small implementable change towards a more plant-based diet can promote cardiovascular health at the population level. Moreover, the transition to a more plant-based diet benefits public health beyond cardiovascular health, as it may also reduce the risk of other chronic diseases such as type 2 diabetes and several cancers [7]. These findings support global public health strategies towards more plant-based diets that are essential for human and planetary health.

## Supplementary Information

Below is the link to the electronic supplementary material.

Supplementary file1 (DOCX 53 KB)

## Data Availability

The data are available on request through the Findata permit procedure at https://www.findata.fi/en/.
