# Supplementary material for: Partial substitution of red or processed meat with plant-based foods and the risk of cardiovascular disease
Source: Eur J Epidemiol. 2025 May 12;40(5):517–25. doi: 10.1007/s10654-025-01232-x (PMC12170763; doi:10.1007/s10654-025-01232-x)
Supplement: Supplementary file 1 — Supplementary file1 (DOCX 53 KB) [file 10654_2025_1232_MOESM1_ESM.docx]

Supplementary material

Title: Partial substitution of red or processed meat with plant-based foods and the risk of cardiovascular disease

Journal: European Journal of Epidemiology

Authors: Meri Simojoki*, Niina E Kaartinen, Mirkka Maukonen, Kennet Harald, Heli Tapanainen, Demetrius Albanes, Johan G Eriksson, Pekka Jousilahti, Seppo Koskinen, Anne-Maria Pajari, Satu Männistö

*Corresponding author:

Finnish Institute for Health and Welfare (THL), P.O. Box 30, 00271, Helsinki, Finland

meri.simojoki@thl.fi

This supplementary material includes six Online Resources:

(1) a table of foods included in each food group used in the substitution analyses

(2) a table of pooled associations between the consumption of red meat, processed meat and the plant-based substitutes, and cardiovascular disease (CVD) risk

(3) a table of the leave-one-out model for studying the impact of partial substitution of red meat or processed meat with plant-based foods on cardiovascular disease risk

(4) a table of pooled associations between partial substitutions of red meat (100 g/day) or processed meat (50 g/day) with legumes, vegetables, fruits, whole grain cereals, or a combination of these, and cardiovascular disease risk in men and women with a median follow-up of 12.7 years

(5) a table of pooled associations between partial substitutions of red meat (100 g/week) or processed meat (50 g/week) with legumes, vegetables, fruits, whole grain cereals, or a combination of these, and cardiovascular disease (CVD) risk in men and women with a median follow-up of 12.7 years: sensitivity analyses

(6) a table of pooled associations between partial substitutions of red meat (100 g/week) or processed meat (50 g/week) with legumes, vegetables, fruits, whole grain cereals, or a combination of these, and cardiovascular disease risk in men and women with a shortened follow-up of 7.9 years.

Online Resource 1. Foods included in each food group used in the substitution analyses

| Food group^a^ | Included foods |
| --- | --- |
| Red meat | Beef, pork, lamb, game |
| Processed meat | Sausages (e.g. fresh sausages, frankfurters), cold cuts (e.g. smoked ham) ^b^ |
| Legumes | All types commonly used in Finland such as beans, green peas, soya |
| Vegetables | Cabbages, leaf vegetables, nuts and seeds^c^, mushrooms, onions, root vegetables^d^, vegetable fruits |
| Fruits | Fruits (e.g. citrus fruits, apples) and berries (e.g. blueberry, strawberry) |
| Whole grain cereals | Rye, oat, and barley (e.g. rye bread, rolled oats) ^e^ |
| Combined plant-based foods | Legumes, vegetables, fruits, whole grain cereals |

^a^Defined by the food grouping in the Finnish National Food Composition Database Fineli® [24]

^b^Sausages and cold cuts made of all kinds of meat

^c^Nuts and seeds included in vegetables because their consumption is low in Finland [5]

^d^Potatoes excluded

^e^Wheat was not included in whole grain cereals because we were unable to separate whole wheat from refined wheat. Combination of rye, oat, and barley has been shown to correspond well (r=0.99) to total whole grain intake in Finnish adults [25].

Online Resource 2. Pooled associations between the consumption of red meat, processed meat and the plant-based substitutes, and cardiovascular disease (CVD) risk

|  | Quintile 1 | Quintile 3 | Quintile 5 | P_trend_ | P_het_^a^ |
| --- | --- | --- | --- | --- | --- |
| Red meat |  |  |  |  |  |
| Median (IQR^b^), g/day | 33 (14) | 67 (8) | 123 (42) | - | - |
| CVD cases, n | 2133 | 2306 | 1938 | - | - |
| Model 1^c^, HR (95% CI)^d^ | 1.00 | 0.98 (0.92, 1.04) | 1.00 (0.93, 1.06) | 0.87 | 0.66 |
| Model 2^e^, HR (95% CI)^d^ | 1.00 | 0.97 (0.92, 1.03) | 0.99 (0.93, 1.06) | 0.95 | 0.68 |
| Processed meat |  |  |  |  |  |
| Median (IQR^b^), g/day | 15 (10) | 51 (10) | 130 (60) | - | - |
| CVD cases, n | 1562 | 2369 | 2536 | - | - |
| Model 1^c^, HR (95% CI)^d^ | 1.00 | 1.09 (0.95, 1.26) | 1.22 (0.96, 1.56) | 0.11 | 0.023 |
| Model 2^e^, HR (95% CI)^d^ | 1.00 | 1.05 (0.92, 1.20) | 1.11 (0.87, 1.41) | 0.47 | 0.064 |
| Legumes |  |  |  |  |  |
| Median (IQR^b^), g/day | 1 (2) | 6 (1) | 17 (13) | - | - |
| CVD cases, n | 2864 | 2295 | 1162 | - | - |
| Model 1^c^, HR (95% CI)^d^ | 1.00 | 0.98 (0.92, 1.03) | 0.97 (0.87, 1.08) | 0.89 | 0.30 |
| Model 2^e^, HR (95% CI)^d^ | 1.00 | 0.99 (0.93, 1.04) | 0.99 (0.91, 1.07) | 0.71 | 0.31 |
| Vegetables^f^ |  |  |  |  |  |
| Median (IQR^b^), g/day | 45 (24) | 132 (27) | 361 (180) | - | - |
| CVD cases, n | 3219 | 2436 | 794 | - | - |
| Model 1^c^, HR (95% CI)^d^ | 1.00 | 0.83 (0.67, 1.01) | 0.76 (0.65, 0.88) | <0.001 | 0.65 |
| Model 2^e^, HR (95% CI)^d^ | 1.00 | 0.88 (0.75, 1.03) | 0.85 (0.76, 0.94) | <0.001 | 0.87 |
| Fruits |  |  |  |  |  |
| Median (IQR^b^), g/day | 31 (26) | 127 (27) | 348 (181) | - | - |
| CVD cases, n | 2620 | 2408 | 1367 | - | - |
| Model 1^c^, HR (95% CI)^d^ | 1.00 | 0.67 (0.48, 0.95) | 0.61 (0.44, 0.85) | 0.023 | 0.001 |
| Model 2^e^, HR (95% CI)^d^ | 1.00 | 0.75 (0.54, 1.05) | 0.74 (0.57, 0.97) | 0.11 | 0.14 |
| Whole grain cereals^g^ |  |  |  |  |  |
| Median (IQR^b^), g/day | 28 (19) | 86 (13) | 177 (55) | - | - |
| CVD cases, n | 1734 | 2107 | 2766 | - | - |
| Model 1^c^, HR (95% CI)^d^ | 1.00 | 0.97 (0.91, 1.04) | 0.94 (0.88, 1.01) | 0.076 | 0.48 |
| Model 2^e^, HR (95% CI)^d^ | 1.00 | 1.04 (0.89, 1.23) | 0.94 (0.88, 1.00) | 0.075 | 0.88 |

^a^P for heterogeneity from Q-statistics between pooled cohorts. Adjusted as Model 2.

^b^Interquartile ranges

^c^Model 1: adjusted for age (years, continuous), sex, and energy intake (kJ/day, continuous).

^d^Hazard ratio and 95% confidence interval

^e^Model 2: adjusted for Model 1 + education (tertiles by birth year), smoking (never, former, current), leisure-time physical activity (passive, somewhat active, active), body mass index (kg/m^2^, continuous), systolic blood pressure (mmHg, continuous), diastolic blood pressure (mmHg, continuous), total serum cholesterol (mmol/l, continuous), alcohol consumption (as ethanol, g/day, continuous).

^f^Nuts and seeds included, legumes and potatoes excluded

^g^Rye, oat, and barley, the combination of which has been shown to correspond well (r=0.99) to total whole grain intake in Finnish adults [25].

Online Resource 3. The leave-one-out model for studying the impact of partial substitution of red meat or processed meat with plant-based foods on cardiovascular disease risk

| Model expression^a^ | Model parameters |
| --- | --- |
|  | A = consumption of the substitute (legumes, vegetables, fruits, whole grain cereals, or a combination of these) |
|  | B = consumption of the food that is being substituted (red meat or processed meat) |
| f(Y) = α_1_A + α_2_(A + B) + confounders | α_1_ = the parameter^b^ for the substitution effect (substituting A for B) |
|  | α_2_ = coefficient^b^ for the sum variable consisting of the substitute and the food that is being substituted (red meat or processed meat) |

^a^Modified from Song et al. 2018 [28]

^b^Regression coefficients are calculated by Cox proportional hazards multivariate models

Online Resource 4. Pooled associations between partial substitutions of red meat (100 g/day) or processed meat (50 g/day) with legumes, vegetables, fruits, whole grain cereals, or a combination of these, and cardiovascular disease risk in men and women with a median follow-up of 12.7 years

|  | Men | |  | Women | | | |  |  |
| --- | --- | --- | --- | --- | --- | --- | --- | --- | --- |
|  | Model 1^a^ | Model 2^b^ |  | Model 1^a^ | Model 2^b^ | | |  |  |
|  | HR (95% CI)^c^ | HR (95% CI)^c^ | P_het_^d^ | HR (95% CI)^c^ | HR (95% CI)^c^ | | | P_het_^d^ |  |
| Substitution of red meat (100 g/day) with |  |  |  |  |  | | |  |  |
| Legumes^e^, 100 g/day | 0.78 (0.42, 1.45) | 0.88 (0.53, 1.46) | 0.24 | 1.79 (0.99, 3.24) | 1.97 (1.07, 3.61)* | | | 0.50 |  |
| Vegetables^f^, 100 g/day | 0.85 (0.77, 0.94)** | 0.94 (0.83, 1.07) | 0.27 | 0.89 (0.74, 1.07) | 0.92 (0.76, 1.11) | | | 0.67 |  |
| Fruits, 100 g/day | 0.94 (0.89, 1.00) | 0.97 (0.92, 1.04) | 0.80 | 0.83 (0.70, 1.00)* | 0.87 (0.72, 1.04) | | | 0.80 |  |
| Whole grain cereals^g^, 100 g/day | 1.00 (0.94, 1.07) | 1.00 (0.94, 1.07) | 0.92 | 0.79 (0.58, 1.06) | 0.85 (0.62, 1.15) | | | 0.95 |  |
| Legumes, vegetables, fruits, and whole grain  cereals, 100 g/day | 0.94 (0.88, 1.00)* | 0.97 (0.91, 1.03) | 0.77 | 0.85 (0.71, 1.02) | 0.88 (0.74, 1.06) | | | 0.75 |  |
|  | |  |  |  | |  |  | | |
| Substitution of processed meat (50 g/day) with | |  |  |  | |  |  | | |
| Legumes^e^, 50 g/day | 0.84 (0.65, 1.10) | 0.89 (0.73, 1.08) | 0.41 | 1.63 (0.90, 2.95) | 1.69 (0.94, 3.03) | | | 0.048 |  |
| Vegetables^f^, 50 g/day | 0.90 (0.88, 0.92)*** | 0.93 (0.91, 0.96)*** | 0.95 | 0.90 (0.81, 0.99)* | 0.91 (0.82, 1.02) | | | 0.061 |  |
| Fruits, 50 g/day | 0.90 (0.85, 0.96)** | 0.95 (0.92, 1.00)* | 0.50 | 0.89 (0.80, 0.98)* | 0.91 (0.81, 1.01) | | | 0.053 |  |
| Whole grain cereals^g^, 50 g/day | 0.95 (0.89, 1.01) | 0.99 (0.96, 1.01) | 0.89 | 0.88 (0.75, 1.03) | 0.93 (0.79, 1.09) | | | 0.18 |  |
| Legumes, vegetables, fruits, and whole grain  cereals, 50 g/day | 0.91 (0.86, 0.97)** | 0.96 (0.92, 1.00)* | 0.50 | 0.89 (0.80, 0.98)* | 0.91 (0.82, 1.01) | | | 0.064 |  |

^a^Model 1: adjusted for age (years, continuous) and energy intake (kJ/day, continuous)

^b^Model 2: adjusted for Model 1 + education (tertiles by birth year), smoking (never, former, current), leisure-time physical activity (passive, somewhat active, active), body mass index (kg/m^2^, continuous), systolic blood pressure (mmHg, continuous), diastolic blood pressure (mmHg, continuous), total serum cholesterol (mmol/l, continuous), hormone replacement therapy (only in women) (ever, never), alcohol consumption (as ethanol, g/day, continuous)

^c^Hazard ratio and 95% confidence interval

^d^P for heterogeneity from Q-statistics between pooled cohorts. Adjusted as Model 2.

^e^Daily consumption of red and processed meat is much higher than that of legumes in Finnish adults, and therefore these results should be interpreted with caution [5, 28].

^f^Nuts and seeds included, legumes and potatoes excluded

^g^Rye, oat, and barley, the combination of which has been shown to correspond well (r=0.99) to total whole grain intake in Finnish adults [25].

*P<0.05, **P<0.01, ***P<0.001

Online Resource 5. Pooled associations between partial substitutions of red meat (100 g/week) or processed meat (50 g/week) with legumes, vegetables, fruits, whole grain cereals, or a combination of these, and cardiovascular disease (CVD) risk in men and women with a median follow-up of 12.7 years: sensitivity analyses

|  | Men | | Women | |
| --- | --- | --- | --- | --- |
|  | Sensitivity analysis 1^a^  HR (95% CI)^c^ | Sensitivity analysis 2^b^  HR (95% CI)^c^ | Sensitivity analysis 1^a^  HR (95% CI)^c^ | Sensitivity analysis 2^b^  HR (95% CI)^c^ |
| Substitution of red meat (100 g/week) with |  |  |  |  |
| Legumes^d^, 100 g/week | 0.98 (0.92, 1.04) | 1.00 (0.93, 1.08) | 1.12 (1.02, 1.22)* | 1.13 (1.03, 1.23)* |
| Vegetables^e^, 100 g/week | 0.99 (0.98, 1.00) | 0.99 (0.98, 1.00) | 0.99 (0.96, 1.02) | 0.99 (0.96, 1.02) |
| Fruits, 100g/week | 1.00 (0.99, 1.01) | 1.00 (0.99, 1.01) | 0.98 (0.95, 1.01) | 0.98 (0.96, 1.01) |
| Whole grain cereals^f^, 100 g/week | 1.00 (0.99, 1.01) | 1.00 (0.99, 1.01) | 0.97 (0.93, 1.02) | 0.98 (0.94, 1.03) |
| Legumes, vegetables, fruits, and whole grain cereals,  100 g/week | 1.00 (0.99, 1.00) | 0.99 (0.99, 1.00) | 0.98 (0.96, 1.01) | 0.99 (0.96, 1.01) |
|  |  |  |  |  |
| Substitution of processed meat (50 g/week) with |  |  |  |  |
| Legumes^d^, 50 g/week | 0.99 (0.96, 1.01) | 0.99 (0.97, 1.02) | 1.05 (1.01, 1.09)* | 1.09 (0.99, 1.20) |
| Vegetables^e^, 50 g/week | 1.00 (0.99, 1.00)* | 1.00 (0.99, 1.00)* | 0.99 (0.97, 1.00) | 0.99 (0.98, 1.01) |
| Fruits, 50 g/week | 1.00 (0.99, 1.00) | 1.00 (0.99, 1.00) | 0.99 (0.97, 1.00) | 0.99 (0.98, 1.01) |
| Whole grain cereals^f^, 50 g/week | 1.00 (0.99, 1.00) | 1.00 (1.00, 1.00) | 0.99 (0.97, 1.02) | 0.99 (0.97, 1.02) |
| Legumes, vegetables, fruits, and whole grain cereals,  50 g/week | 0.99 (0.99, 1.00)* | 1.00 (0.99, 1.00)* | 0.99 (0.97, 1.00) | 0.99 (0.98, 1.01) |

^a^Sensitivity analysis 1: Excluding participants who consumed red meat ˂100 g/week (n=779) and processed meat ˂50 g/week (n=1644).

^b^Sensitivity analysis 2: Excluding participants diagnosed with CVD in the first two years of follow-up due to concerns about reverse causation: ATBC (n=910), Health 2000 (n=115), HBCS (n=11), DILGOM 2007 (n=29) and FINRISK 2012 (n=13).

^c^Hazard ratio and 95% confidence interval. Adjusted as Model 2: age (years, continuous), energy intake (kJ/day, continuous), education (tertiles by birth year), smoking (never, former, current), leisure-time physical activity (passive, somewhat active, active), body mass index (kg/m^2^, continuous), systolic blood pressure (mmHg, continuous), diastolic blood pressure (mmHg, continuous), total serum cholesterol (mmol/l, continuous), hormone replacement therapy (only in women) (ever, never), alcohol consumption (as ethanol, g/day, continuous).

^d^Daily consumption of red and processed meat is much higher than that of legumes in Finnish adults, and therefore these results should be interpreted with caution [5, 28].

^e^Nuts and seeds included, legumes and potatoes excluded

^f^Rye, oat, and barley, the combination of which has been shown to correspond well (r=0.99) to total whole grain intake in Finnish adults [25].

*P<0.05

Online Resource 6. Pooled associations between partial substitutions of red meat (100 g/week) or processed meat (50 g/week) with legumes, vegetables, fruits, whole grain cereals, or a combination of these, and cardiovascular disease risk in men and women with a shortened follow-up of 7.9 years

|  | Men | |  | Women | | | |  |  |
| --- | --- | --- | --- | --- | --- | --- | --- | --- | --- |
|  | Model 1^a^ | Model 2^b^ |  | Model 1^a^ | Model 2^b^ | | |  |  |
|  | HR (95% CI)^c^ | HR (95% CI)^c^ | P_het_^d^ | HR (95% CI)^c^ | HR (95% CI)^c^ | | | P_het_^d^ |  |
| Substitution of red meat (100 g/week) with |  |  |  |  |  | | |  |  |
| Legumes^e^, 100 g/week | 0.94 (0.86, 1.04) | 0.96 (0.88, 1.04) | 0.37 | 1.05 (0.94, 1.17) | 1.06 (0.95, 1.18) | | | 0.83 |  |
| Vegetables^f^, 100 g/week | 0.98 (0.96, 0.99)** | 0.99 (0.97, 1.01) | 0.39 | 0.97 (0.94, 1.00)* | 0.97 (0.94, 1.00) | | | 0.78 |  |
| Fruits, 100 g/week | 1.00 (0.98, 1.01) | 1.00 (0.99, 1.02) | 0.40 | 0.96 (0.93, 0.99)** | 0.96 (0.93, 1.00)* | | | 0.85 |  |
| Whole grain cereals^g^, 100 g/week | 1.00 (0.99, 1.02) | 1.01 (0.99, 1.02) | 0.80 | 0.92 (0.85, 1.00) | 0.94 (0.87, 1.01) | | | 0.30 |  |
| Legumes, vegetables, fruits, and whole grain  cereals, 100 g/week | 1.00 (0.98, 1.01) | 1.00 (0.99, 1.01) | 0.52 | 0.96 (0.93, 0.99)* | 0.97 (0.94, 1.00)* | | | 0.82 |  |
|  | |  |  |  | |  |  | | |
| Substitution of processed meat (50 g/week) with | |  |  |  | |  |  | | |
| Legumes^e^, 50 g/week | 0.96 (0.92, 1.00)* | 0.96 (0.93, 1.00)* | 0.44 | 1.03 (0.98, 1.08) | 1.04 (0.99, 1.08) | | | 1.00 |  |
| Vegetables^f^, 50 g/week | 0.98 (0.98, 0.99)*** | 0.99 (0.99, 1.00)*** | 0.91 | 0.97 (0.96, 0.99)** | 0.98 (0.96, 0.99)** | | | 0.24 |  |
| Fruits, 50 g/week | 0.99 (0.97, 1.00)** | 0.99 (0.99, 1.00) | 0.58 | 0.97 (0.96, 0.99)** | 0.98 (0.96, 0.99)** | | | 0.23 |  |
| Whole grain cereals^g^, 50 g/week | 0.99 (0.97, 1.00) | 1.00 (0.99, 1.01) | 0.61 | 0.97 (0.94, 1.00)* | 0.98 (0.95, 1.01) | | | 0.29 |  |
| Legumes, vegetables, fruits, and whole grain  cereals, 50 g/week | 0.99 (0.98, 1.00)** | 0.99 (0.99, 1.00) | 0.49 | 0.97 (0.96, 0.99)** | 0.98 (0.96, 0.99)** | | | 0.17 |  |

^a^Model 1: adjusted for age (years, continuous) and energy intake (kJ/day, continuous)

^b^Model 2: adjusted for Model 1 + education (tertiles by birth year), smoking (never, former, current), leisure-time physical activity (passive, somewhat active, active), body mass index (kg/m^2^, continuous), systolic blood pressure (mmHg, continuous), diastolic blood pressure (mmHg, continuous), total serum cholesterol (mmol/l, continuous), hormone replacement therapy (only in women) (ever, never), alcohol consumption (as ethanol, g/day, continuous)

^c^Hazard ratio and 95% confidence interval

^d^P for heterogeneity from Q-statistics between pooled cohorts. Adjusted as Model 2.

^e^Daily consumption of red and processed meat is much higher than that of legumes in Finnish adults, and therefore these results should be interpreted with caution [5, 28].

^f^Nuts and seeds included, legumes and potatoes excluded

^g^Rye, oat, and barley, the combination of which has been shown to correspond well (r=0.99) to total whole grain intake in Finnish adults [25].

*P<0.05, **P<0.01, ***P<0.001
